# Parallel Epigenomic and Transcriptomic Responses to Viral Infection in Honey Bees (*Apis mellifera*)

**DOI:** 10.1371/journal.ppat.1004713

**Published:** 2015-03-26

**Authors:** David A. Galbraith, Xingyu Yang, Elina Lastro Niño, Soojin Yi, Christina Grozinger

**Affiliations:** 1 Department of Entomology, Center for Pollinator Research, Pennsylvania State University, University Park, Pennsylvania, United States of America; 2 School of Biology, Georgia Institute of Technology, Atlanta, Georgia, United States of America; Stanford University, UNITED STATES

## Abstract

Populations of honey bees are declining throughout the world, with US beekeepers losing 30% of their colonies each winter. Though multiple factors are driving these colony losses, it is increasingly clear that viruses play a major role. However, information about the molecular mechanisms mediating antiviral immunity in honey bees is surprisingly limited. Here, we examined the transcriptional and epigenetic (DNA methylation) responses to viral infection in honey bee workers. One-day old worker honey bees were fed solutions containing Israeli Acute Paralysis Virus (IAPV), a virus which causes muscle paralysis and death and has previously been associated with colony loss. Uninfected control and infected, symptomatic bees were collected within 20–24 hours after infection. Worker fat bodies, the primary tissue involved in metabolism, detoxification and immune responses, were collected for analysis. We performed transcriptome- and bisulfite-sequencing of the worker fat bodies to identify genome-wide gene expression and DNA methylation patterns associated with viral infection. There were 753 differentially expressed genes (FDR<0.05) in infected versus control bees, including several genes involved in epigenetic and antiviral pathways. DNA methylation status of 156 genes (FDR<0.1) changed significantly as a result of the infection, including those involved in antiviral responses in humans. There was no significant overlap between the significantly differentially expressed and significantly differentially methylated genes, and indeed, the genomic characteristics of these sets of genes were quite distinct. Our results indicate that honey bees have two distinct molecular pathways, mediated by transcription and methylation, that modulate protein levels and/or function in response to viral infections.

## Author Summary

Honey bees are a critical pollinator of a wide variety of agricultural crops, but beekeepers experience heavy annual losses of honey bee colonies. Several factors are associated with colony losses, in particular infection with Israeli Acute Paralysis Virus (IAPV). Despite the importance of viruses to honey bee health, our understanding of the molecular mechanisms mediating host-pathogen interactions is limited. Here, we characterized the epigenomic and transcriptomic responses of honey bees to short term (<24 hour) IAPV infection. We found significant expression differences in 753 genes between infected and control bees, including genes involved in immune and epigenetic pathways. However, IAPV-regulated genes did not overlap significantly with genes that respond to bacterial or microsporidian infection, suggesting that honey bees utilize distinct pathways for different immune challenges. Additionally, DNA methylation status of 156 genes changed significantly, including several genes that have been linked to antiviral immune responses in humans. Interestingly, there was no significant overlap between the differentially methylated and differentially expressed genes, suggesting that honey bees may possess a parallel genomic mechanisms to respond to viral infection.

## Introduction

Honey bee populations are in decline throughout the world [1]. A recent survey found that US beekeepers lose 30% of their colonies annually [2]. Because honey bees are critical pollinators of our agricultural crops and over 70% of major global food crops, including fruits, vegetables and nuts, benefit from honey bees and other pollinators [3], these losses have substantial implications for the sustainability of our agricultural industry. Several factors are thought to contribute to these declines, including pathogens, parasites, habitat loss, poor nutrition due to monocropping systems, and pesticide use [1]. Furthermore, several of these factors appear to interact synergistically; for example, the titers and impacts of viruses increase in bees infested with *Varroa* mites [4], fed poor diets [5] or exposed to pesticides [6]. Honey bees have several viruses, with more than 20 identified thus far [7,8]. These viruses can have a wide range of impacts, from no obvious symptoms to developmental defects [9], altered learning and memory [10], and loss of muscle coordination and premature death [11]. In several studies, increasing numbers of viruses or viral titers have been linked to colony losses [12–14]. There is currently a wealth of information on the pleiotropic effects of viral infection in honey bees, but information about the molecular and physiological responses of honey bees to viral infections, especially on the genome-wide scale, is surprisingly limited.

One of the viruses that has been implicated in the decline of honey bees is Israeli Acute Paralysis Virus (IAPV). IAPV is a positive sense RNA virus in the family *Dicistroviridae* [15]. Infection of honey bee pupae results in cessation of development and altered expression of several candidate genes involved in ribosomal biogenesis, though the impacts of infection can be quite variable [16]. Infection of caged adult bees results in decreased locomotion, muscle spasms, and increased mortality, with 80% of infected bees dying prematurely[11]. Though IAPV infection was originally associated with the phenomenon known as "Colony Collapse Disorder" [17,18], subsequent studies failed to provide a strong association between IAPV infection and colony losses associated with this syndrome [15,19,20]. However, a recent study demonstrated that IAPV is more prevalent in weak colonies which fail to survive the winter [21]. Thus, though the role of IAPV in colony losses is not well understood, it seems likely that presence of this virus reduces colony strength and survival.

The molecular mechanisms involved in the antiviral responses in insects have been best studied in *Drosophila melanogaster*. Antiviral response appear to be mediated by two primary pathways, one involving JAK-STAT (Janus kinase—signal transducers and activators of transcription) and the other RNA interference (RNAi) [22]. The JAK-STAT pathway uses cytokine molecules to activate the signaling cascade upon binding to receptors on the cell surface. The activated receptor then causes a dimerization of Stat transcription factors, which enter the nucleus and binds to downstream viral response genes with Stat binding sites in their promoter regions. The RNAi pathway functions by cleaving double stranded RNA (dsRNA) into small fragments, which are used to target other mRNA transcripts with the same sequence, and thereby preventing the translation from mRNA to protein and effectively decreasing the activity of the particular gene. Mutant *D*. *melanogaster* that do not possess a functional RNAi system have increased mortality and higher viral titers, suggesting that this pathway is an essential part of the antiviral immunity in flies [23]. Previous studies have suggested that the RNAi pathway may also be involved in the honey bee antiviral response, since honey bees show decreased viral titers after treatment with virus specific dsRNA and non-specific dsRNA[11,24–26]. However, though these studies demonstrate the principle that viral titers can be reduced through RNAi, they did not demonstrate that transcriptional regulation of the RNAi pathway is an antiviral response in honey bees. Other recent studies have examined transcriptional responses to Deformed Wing Virus in pre-adult stages[27], three days post infection with a model virus (Sindbis) in young worker bees[24], and in older bees chronically infected with IAPV[21], but the global transcriptional and epigenetic responses to acute viral infections in adult bees has not been investigated.

Epigenetic mechanisms, such as RNAi and DNA methylation, have been shown to play a role in mediating antiviral defenses in vertebrates and plants through post translational modification (protein folding, RNAi, and alternative splicing) [28–30]. Epigenetic mechanisms enable modification of function or expression of DNA, RNA, or proteins without changing the original DNA sequence. In humans and plants, viral infection (of both integrating and non-integrating viruses) has the ability to alter the host DNA methylation patterns[31–36]. Though the majority of insect immune studies have involved *D*. *melanogaster*, it does not possess a full complement of DNA methylation enzymes, and thus while DNA methylation is present, it is at very low levels[37,38]. However, many other insect species, including honey bees, do possess a fully functional DNA methylation system [39], and thus DNA methylation may represent an important antiviral response in insects as well.

In insects, most methylation occurs in the gene body (from the transcriptional start site to the end of transcription, encompassing both exons and introns only), and how this impacts gene function is still under investigation[40,41]. DNA methylation involves the addition of a methyl group (CH_3_) to the 5^th^ carbon in the cytosine pyrimidine ring and primarily occurs within CpG (cytosine phosphate guanine) dinucleotides, but it also occurs to a much lesser extent in CHG and CHH sequence contexts (H = A, C, or T) [42]. Methylated DNA can recruit histone modifying proteins that then alter the structure of the chromatin [42]. This change in DNA packaging can have different effects depending on the genomic context in which it occurs. If DNA methylation occurs within or near a promoter region, the outcome is typically transcriptional silencing [42]. The function of DNA methylation within the gene body is less clear; it has been proposed to function in alternative splicing [43], phenotypic plasticity [44], and suppression of transcriptional noise [45,46]. More research is needed to definitively characterize the function of intragenic DNA methylation.

Here, we examine the transcriptional and epigenetic (DNA methylation) responses of young worker honey bees to acute (20–24 h) infection with IAPV. In control and virus infected workers, we **(1)** characterized genome-wide gene expression patterns in the fat body tissues, the major sites of metabolism, detoxification and immune responses in insects **(2)** characterized associated genome-wide DNA methylation patterns, and **(3)** determined if there is a correlation between gene expression and DNA methylation patterns. Our studies provide insight into the transcriptional and epigenetic mechanisms underlying both antiviral responses in honey bees and the pleiotropic effects of these viruses, while also allowing us to examine in detail the relationship between gene expression and DNA methylation.

## Methods

### Identification of IAPV-free experimental colonies

Colonies of honey bees were maintained at apiaries at Penn State using standard commercial apiculture practices. Colonies headed by single-drone inseminated (SDI) queens (obtained from Glenn Apiaries, Fallbrook, CA) were screened to identify colonies that were free of detectable IAPV infections and had, overall, the lowest numbers and titers of viruses. RNA was isolated from the pooled abdomens of five individuals from each SDI colony using an RNeasy (Qiagen, Valencia, CA) protocol. Using a standard protocol[47], 150 ng of RNA was converted to cDNA and quantitative real time PCR (qRT-PCR) was performed using primers specific for the following viruses: Black Queen Cell Virus (BQCV), Deformed Wing Virus (DWV), Israeli Acute Paralysis Virus (IAPV), and Kashmir Bee Virus (KBV) (see S1B Table for a listing of the primers used and the associated references). Workers from the colony found to be IAPV-free and with the lowest total viral loads were selected and used in subsequent analyses. Note that the lack of IAPV viral infection and reduced levels of other viruses in control bees was confirmed by qRT-PCR and by scanning for viral transcripts in the transcriptomic data (S1C&D Table).

### Treatments

Honeycomb frames of emerging brood were collected placed in a 34°C, 50%RH incubator overnight. Emerged bees (<24 hours old) were collected. Individual workers were fed extracts of virus-infected bees (which contained IAPV, DWV, BQCV, KBV and SBV; bees were obtained from D. Cox-Foster, Penn State) in a 50%-sucrose solution while control bees were fed a 50%-sucrose control (4 μL/bee). These honey bee viruses currently cannot be produced as pure strains under laboratory conditions, so we modified the protocol from Li et al. [48] to extract the viruses from live bees known to be infected with multiple viruses. The viral extracts were obtained by homogenizing two whole bees in 2ml of molecular grade water. Prior to feeding, the inoculum was dialyzed through a 0.20 μm cell filter to purify the virus extract, as in Hunter et al. and Liu et al [25,49]. Sucrose was added to produce a 50% sucrose solution.

Bees were maintained in Plexiglas cages (10 x 10 x 7 cm) in groups of 45 under red light in a 34.5°C and ∼50% RH incubator. Bees were fed 50% sucrose/water and a 50% MegaBee diet/honey mixture *ad libitum*. Each cage was also provided with 0.1 queen equivalents of queen mandibular pheromone (QMP) to approximate normal colony conditions [50]. Bees were monitored hourly for symptoms of viral infection (trembling, twitching) starting 15 hours post infection. Samples for analysis were taken from control and virus-infected bees that began exhibiting symptoms 20–24 hours after treatment (when they were <48 hours old). Only bees exhibiting the neurological symptoms of a successful viral infection were sampled from the virus-infected treatments.

### Confirmation of viral infection

A subset of control and infected individuals was used to confirm viral infection (or lack thereof, in the case of the controls) using qRT-PCR, as above. RNA was isolated from eviscerated abdomens with attached fat bodies of individual bees using TRIzol Reagent (Invitrogen, Carlsbad, CA). Bees were individually screened for infections with Chronic Bee Paralysis Virus (CBPV), Acute Bee Paralysis Virus (ABPV), Sacbrood Virus (SBV) and IAPV (see S1B Table for primers and associated references). Furthermore, we confirmed the presence of the negative strand of IAPV in the treated bees, demonstrating that the virus was actively replicating (S1 Fig.).

### Sample preparation for RNA-Seq and BS-Seq

Eviscerated abdomens with attached fat bodies from individual bees were dissected in ice-cold RNAlater (Qiagen, Valencia, CA) and excess RNAlater was removed from the samples. RNA was isolated using TRIzol Reagent (Invitrogen, Carlsbad, CA). Samples were then treated with TURBO DNase (Ambion, Austin, TX) to remove DNA contamination. The samples were then pooled into three groups containing three individuals for both treatment and control groups. The quality and concentrations of the RNA pools was verified using a 2100 Bioanalyzer (Agilent, Santa Clara, CA).

DNA was isolated from the fat bodies from a second set of bees using a Gentra Puregene kit (Qiagen, Valencia, CA). DNA quality was assessed by electrophoresis, using 1% Agarose gel, and the concentration was determined using a Qubit 2.0 Fluorometer (Invitrogen, Carlsbad, CA). Due to the large amount of DNA required for BS-Seq, the DNA was pooled to produce a single control (n = 9 bees) and a single treatment sample (n = 9 bees).

### RNA-Seq and BS-Seq

The RNA and DNA samples were sent to Beijing Genomics Institute (BGI)(Shenzhen, China) for library preparation and sequencing. The library preparation (each library was individually tagged for each pool) and Illumina paired end sequencing of the RNA samples and bisulfite conversion and Illumina paired end sequencing of DNA samples was performed by BGI. RNA and DNA Samples were sequenced on two separate lanes of the Illumina Hiseq2000 platform, resulting in paired end reads that were 2x100bp in length for the RNA samples and 2x90bp for the DNA samples. The total number of reads for the RNA-seq samples were between 51,231,170 and 53,823,222, and for the BS-seq, the total number of reads for each sample were between 101,423,954 and 102,213,608. Data generated from these studies has been deposited in NCBI’s Gene Expression Omnibus[51] and are accessible through the GEO Series accession number GSE65659.

### Analysis of RNA-Seq data

Transcriptome sequencing reads were preprocessed using Illumina HCS 1.1 software. We removed adaptor sequences, reads composed of more than 5% unknown nucleotides, and reads with more than 20% of the base qualities under 10. The transcriptome sequencing reads were aligned to the most recent honey bee genome assembly (Amel_4.5) [52] using Tophat[53]. Aligned read counts were imported into R statistical software (http://www.r-project.org). Genes with low read counts (less than 5 reads per gene) were removed. The data was normalized using a trimmed mean of M-values (TMM) method [54]. The DESeq package in R statistical program was used to identify significantly differentially expressed genes [55].

For the gene ontology (GO) analysis, *D*. *melanogaster* orthologs of the differentially expressed genes were uploaded to DAVID Bioinformatics Resources 6.7[56]. The overrepresented GO terms of differentially expressed genes during viral infection were determined using a background list of all genes expressed among the samples. To refine the list of GO terms, the significant terms (FDR < 0.05) that were generated using DAVID Bioinformatics Resources were imported into REVIGO [57], which clusters the GO terms using semantic similarity measures.

### qRT-PCR validation of expression differences

To validate the results of the transcriptomic analysis, we repeated the experiment using a new set of bees. Emerging worker bees were collected and fed either viral extracts in a 50% sucrose solution or a 50% sucrose control solution as before. As an additional control, we also included a bee lysate control, using healthy, non-symptomatic bees, that was extensively washed to remove as much of the virus as possible. The groups were reared under the same conditions as before, and monitored hourly for symptoms of viral infection starting 15 hours post infection. All bees were collected 24 hours after treatment. RNA was extracted as above and qRT-PCR was then used to validate the RNA-seq results (see S1B Table for a listing of the primers and associated references).

### Analysis of BS-Seq data

The bisulfite-converted DNA reads in both control and treated group are aligned to honey bee genome Apimel4.5 using BSMAP [58] Ambiguously mapped reads were filtered with parameter-u and potential PCR duplications were removed by parameter-r. To avoid erroneous annotation, we restricted our analyses to genes that fit the following criteria: 1) mapped by RNA-seq data, 2) have orthologs in *D*. *melanogaster*, and 3) with corresponding assembly in the previous assembly 2.0. Following this filtering step, we examined a total of 12,446 genes.

Methylated CpG sites are determined by the binomial test [59]. The error rate (non-conversion rate and sequencing error) is estimated from the sequencing result of the unmethylated λ phage mixed in our sample [60]. Error rates estimated from these were 0.0041 and 0.0037 from control and treatment groups, respectively. P-values were subsequently corrected for multiple testing [61], and sites with adjusted p-value < 0.1 were considered methylated.

Fractional methylation values were calculated for each CpG site as mCG/CG, where mCG is the number of reads with a methylated cytosine at a CpG site (according to non-conversion) and CG is the total number of reads mapped to the site [41]. Fractional methylation for specific genomic regions (for example, gene body, exon or intron) was calculated as the mean of all CpG fractional methylation values within that region. The degree of CpG depletion, or *CpG*_*O/E*_, is calculated as in Elango et al. [62].

### Differential methylation

The differential methylation level of each CpG site was assessed by a Fisher’s exact test with P-values adjusted for multiple testing [61]. Differential methylation of specific genomic regions (such as gene bodies) was assessed by a generalized linear model with binomial family, similar to [59,63]. In the generalized model, the response vector is the number of methylated and nonmethylated CpGs in specific genomic regions, modeled by two categorical factors, treatment (control or infected) and CpGs locations [59,63].

### Alternative splicing

Transcriptome sequencing reads were mapped to honeybee assembly 4.5 and processed by TopHat[53]. Upon detecting a splice junction, two types of alternative splicing events were summarized by comparing them to the known annotation (S2 Fig.): **I)** Intron retention if a pair of junctions occur inside an annotated exon (intron is spliced in our sample, and retained in the reference sequence) and **II)** Exon skipping if the region between a pair of junction covers one or more annotated exons (exon is skipped in our sample).

## Results and Discussion

### Confirmation of viral infection levels

We used qRT-PCR to examine titers of viruses in our control and treated samples. For DWV, CBPV, and BQCV, in all samples (including both treatment and control) viral RNA was either undetectable or showed very late amplification (average C_t_ > 35), while IAPV titers were detected in the treatment samples only (see S1C Table). We also confirmed the presence of the negative strand of IAPV, demonstrating viral replication in the treatment samples (see S1 Fig.). As another measure to confirm successful viral infection, the reads from the RNA-seq data set were aligned to a panel of viral genomes (see S1D Table for accession numbers). The number of reads mapping to IAPV in the treatment samples were substantially higher than those in the controls (S1D Table). Reads for additional viruses (BQCV, DWV, and KBV) were also present in the samples, but at much lower abundance (over 2000x lower than the IAPV reads in the treated group) and were equally abundant in both control and treated samples. While we screened the transcriptome for the common viruses that are pathogenic to honey bees in North America (S1D Table), it is possible that additional viruses were present that we did not account for.

### Transcriptional response to viral infection

**Fig 1:**
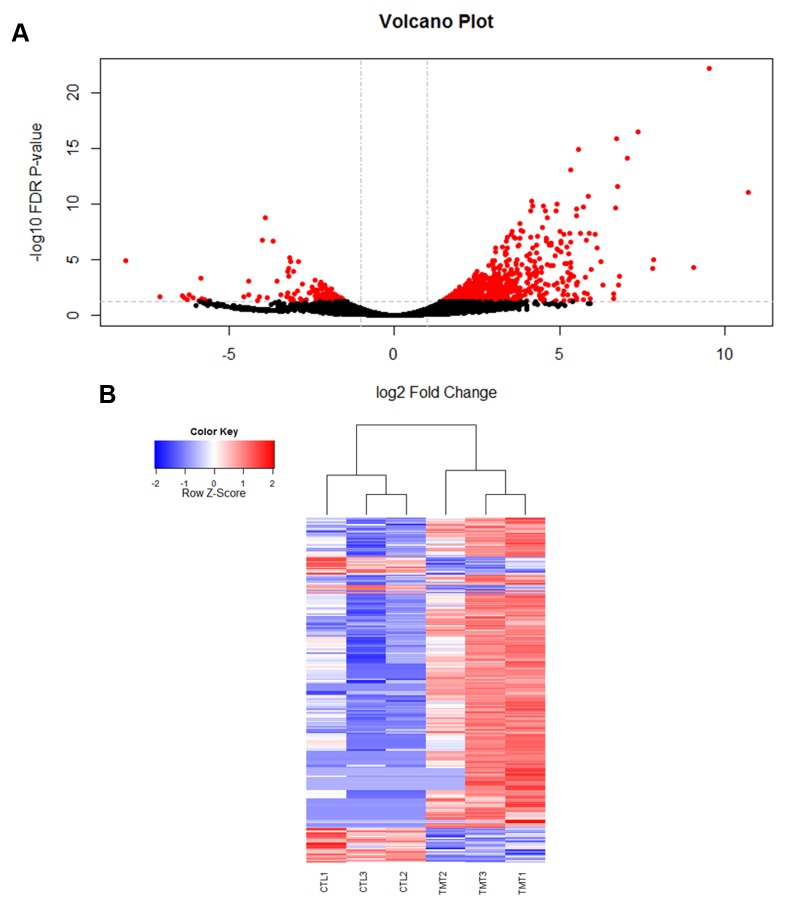
Viral infection with IAPV alters gene expression patterns in honey bee fat bodies. **A)** A volcano plot was created to visualize the amount of significantly differentially expressed genes. The horizontal line represents an FDR p-value of 0.05 and the vertical lines represent a two-fold change in the log of the expression. The red circles indicate genes that are significantly differentially expressed in response to viral infection. 753 genes were found to be significantly differentially expressed (FDR <0.05). **B)** A heat map of the log 2 transformed read counts for the 753 significantly differentially expressed genes was generated. Transcription levels were normalized using a TMM method. Red colors indicate higher levels of gene expression and blue colors indicated lower levels of gene expression relative to the average across the samples. Treatment and control samples are denoted by “TMT” and “CTL” respectively. The heat map was created with R statistical software.

**Fig 2:**
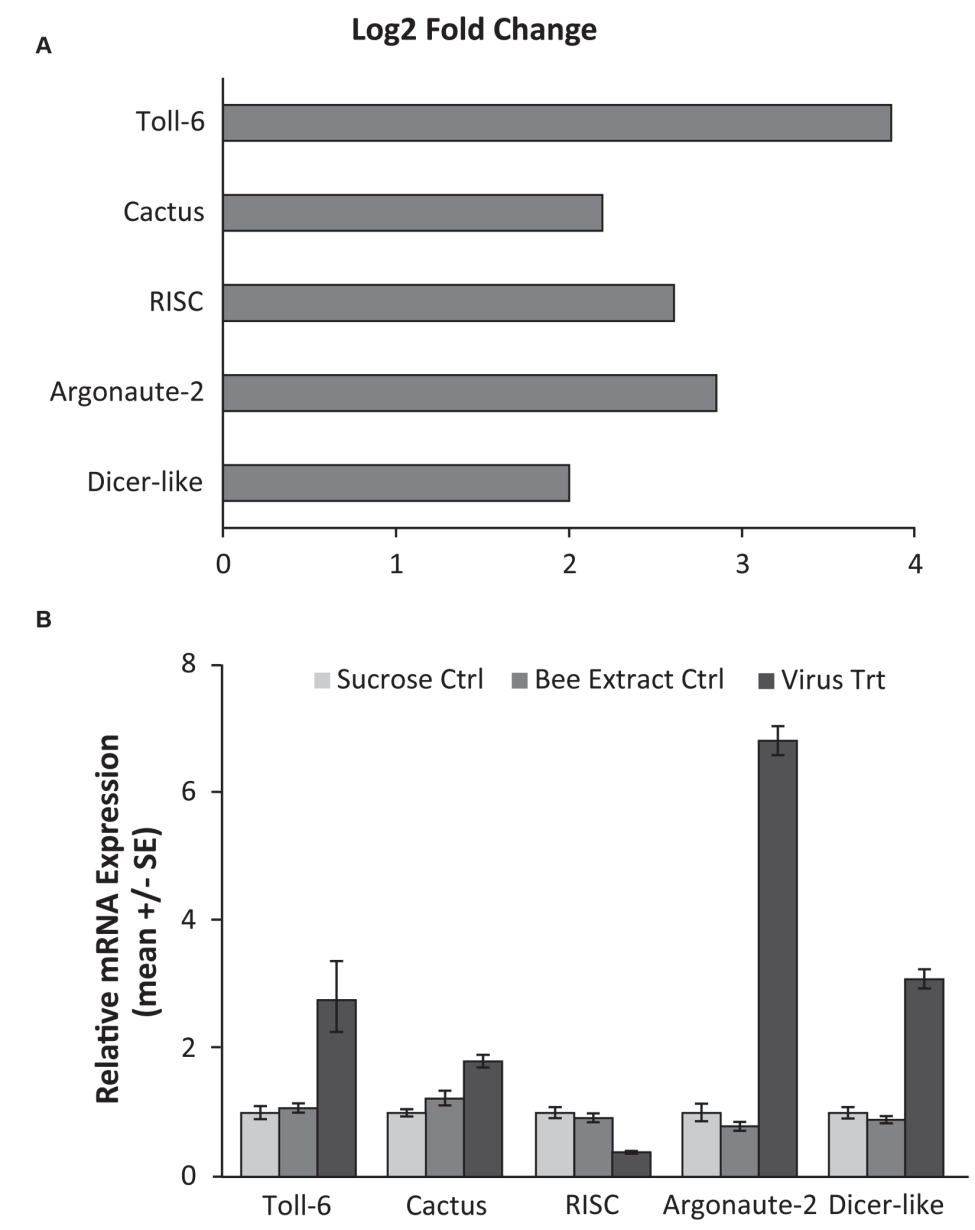
Validation of RNA-seq results for genes in RNAi and Toll pathways. **A)** RNA-seq results, demonstrating the log_2_ fold change (expression in infected vs control groups) of three genes from the RNAi pathway, which has been shown to be used in antiviral responses in Drosophila, as well as two genes from the Toll pathway that have been hypothesized to be implicated in antiviral defense. The log fold change was calculated using the DESeq package in R. **B)** qRT-PCR confirmation of the gene expression in separate bee samples fed either 50% sucrose solution (Sucrose Ctrl), bee extract from individuals with no IAPV infection (Bee Extract Ctrl), and bee extract from individuals with confirmed IAPV infection (Virus Trt)(ANOVA with Tukey HSD posthoc analysis; n = 10 bees/group). Accession numbers and primer sequences can be found in S1B Table.

Using RNA-seq to monitor genome-wide expression patterns in the fat bodies of control and treated bees, we identified 753 genes that were significantly differentially expressed (FDR p-value < 0.05; S1A Table), with 607 genes up-regulated and 146 down-regulated in response to viral infection (Fig. 1A). IAPV-infected and control bees clearly had distinct expression profiles and were clearly separated according to a hierarchical clustering analysis (Fig. 1B). Gene ontology analysis revealed that the genes that were down-regulated were related to response to toxic substances, cofactor metabolic process, glucose catabolic process, and alcohol catabolic process (S1E Table), while genes that were up-regulated were significantly enriched for categories including regulation of MAPK cascade, metabolism, morphogenesis, phosphorylation, autophagic cell death, nucleic acid metabolism, and negative regulation of transcription from RNA polymerase II promoter (S1F Table). Interestingly, transcriptional pausing of RNA polymerase II has been suggested as an innate immune response in *D*. *melanogaster*, priming the host genome by increasing the accessibility of promoter regions associated with virally induced genes, allowing for a more rapid response[64]. Our data also indicate that several core insect immune pathways that have been previously associated with antiviral defense in other insects [22,65,66], including genes from two immune signaling pathways (Toll and Jak-STAT) and the RNAi pathway, are involved in the acute antiviral immune response in honey bees. We found a significant upregulation of multiple representatives of the RNAi pathway (Fig. 2A), including *Argonaute-2* and *Dicer-like* using qRT-PCR. Honey bees encode multiple Dicer proteins (*Dicer-1* and *Dicer-like*), but *Dicer-like* is primarily involved in RNAi[67]. Using qRT-PCR, we confirmed upregulation of genes involved in the RNAi and Toll pathways (Fig. 2B). All but one of the candidate genes (*tarbp2-like*) we tested matched the expression pattern that was seen in the RNA-seq data. This is the first time the RNAi pathway has been implicated in a transcriptional antiviral response in honey bees, though previous studies have demonstrated that feeding with double-stranded RNA constructs, both virus specific and non-specific, (which induces an RNAi response) can reduce viral titers[11,24–26,49].

**Fig 3:**
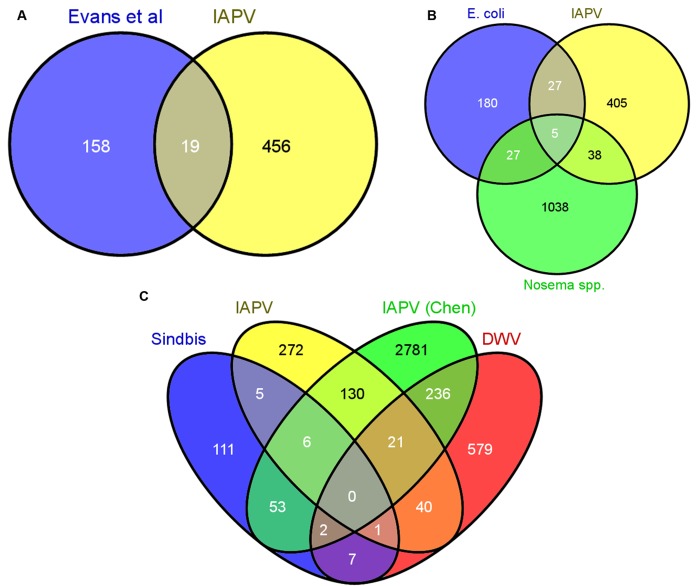
Comparative analysis of transcriptional responses to different immunostimulants. **A)** The 753 differentially expressed genes were compared to the set of canonical immune genes identified in the genome by Evans et al (2006). Only 19 genes were shared between these groups **B)** The differentially expressed genes were compared to genes differentially regulated in response to infection with *E*. *coli* and the microsporidian parasites *Nosema apis* and *Nosema ceranae*. There was little overlap among the groups, indicating that responses to acute viral infections utilize distinct pathways. **C)** Overlap with three previous studies examining transcriptional responses to Sindbis virus[24], IAPV[21] (from Chen et al, 2014), and DWV[27]. For all analyses, the Venn diagram was created using Venny.

Next, we performed a series of comparative studies with previous-genome wide analyses of transcriptional responses to parasite and pathogen infections in honey bees and *D*. *melanogaster*. A comparison of our suite of regulated genes with the 177 genes identified as part of the honey bee immune response pathways in Evans et al. [68] revealed an overlap of only 19 genes (S1G Table), demonstrating that there are many other genes that may play a role in antiviral responses (Fig. 3A). Comparing our gene expression results to an antiviral study in *D*. *melanogaster* by Xu et al. [64], we found an overlap of 29 genes (S1H Table). Interestingly, Xu et al. also found that the transcriptional pausing pathway was involved in the *D*. *melanogaster* acute antiviral response, including upregulation of the gene encoding negative elongation factor (NELF), which plays an important role in transcriptional pausing and was also found to be upregulated in our study. Indeed, of the 29 genes overlapping between our study and Xu et al, there were two nonsignificantly overrepresented GO terms, regulation of transcription and autophagic cell death, further suggesting a link between transcriptional pausing and antiviral response.

Previous studies identified genes differentially regulated in the fat bodies of worker bees injected with saline, beads or *Escherichia coli* bacteria [69] or of worker bees chronically infected with microsporidian (*Nosema spp*.) intestinal parasites [70]. We found little overlap among the genes identified in these studies and those responding to viral infection in our current study (38 for *N*. *spp*. and 27 for *E*. *coli*), and only 5 were present in all three treatments (Fig. 3B and S1I Table). The lack of a significant overlap (p > 0.05, Fisher Exact Test) with either of these two studies suggests that honey bees use different molecular mechanisms to defend themselves against different pathogens. When we compared our gene expression results to three other studies (Fig. 3C, S1I Table) examining transcriptional responses to viral infection in honey bees (DWV, IAPV, and Sindbis Virus (a model virus)), we found a greater degree of overlap, however, there is still variability among the antiviral responses. This variability could be due to differences in antiviral response across life stages or to the different viruses. Notably, of all the studies we compared, the greatest overlap with our results was found with a study examining the effects of chronic IAPV infection on older adult workers[21].

### Characteristics of methylated genes in honey bees

**Fig 4:**
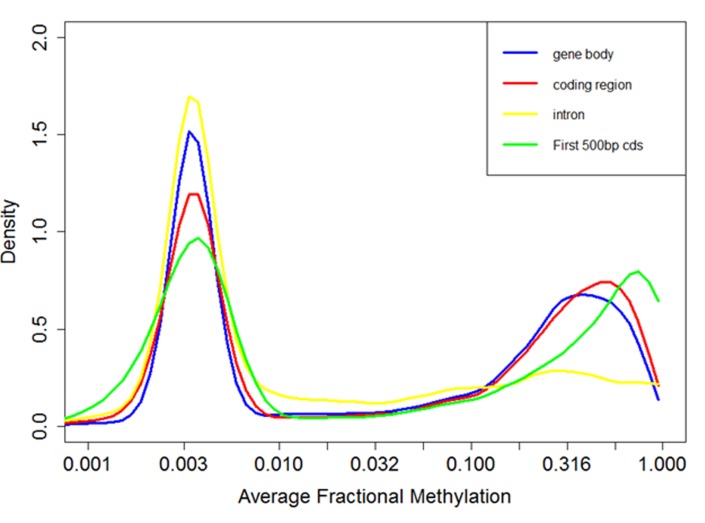
Distribution of fractional methylation levels of genes. Distribution of DNA methylation levels (measured by fractional methylation levels) of genes. The X-axis is drawn in a log-transformed scale with the actual methylation levels indicated. A total of 11,063 genes are grouped into 100 equal size parts. The genes clearly divide into two groups, one with sparse DNA methylation (peak at around 0.03) and the other with relatively high DNA methylation (peak at around 0.6).

In agreement with previous studies[62], we found that DNA methylation is mostly targeted to gene bodies (exons and introns, Fig. 4). Fractional methylation levels of gene bodies exhibit a distinctive bimodal pattern where one group of genes shows little DNA methylation (fractional methylation level peak at 0.03) while the other group of genes shows substantial methylation peak at 0.3 (Fig. 4). This pattern is similar to the results of a previous analysis of experimental data [71] as well as computational predictions [62,72,73]. The bimodal pattern of DNA methylation of genic regions is consistently observed when we restrict our analyses to CpGs occurring only in exons or introns, or in the first 500 bps of a gene (Fig. 4). Based upon this observation, we tentatively refer to genes with fractional methylation levels of exons < 0.03 as ‘non-methylated genes (nonMGs)’, and those greater than 0.03 as ‘methylated genes (MGs)’. Defined this way, most genes remained in the same group (MGs or nonMGs) in the control and treatment groups (see below).

**Fig 5:**
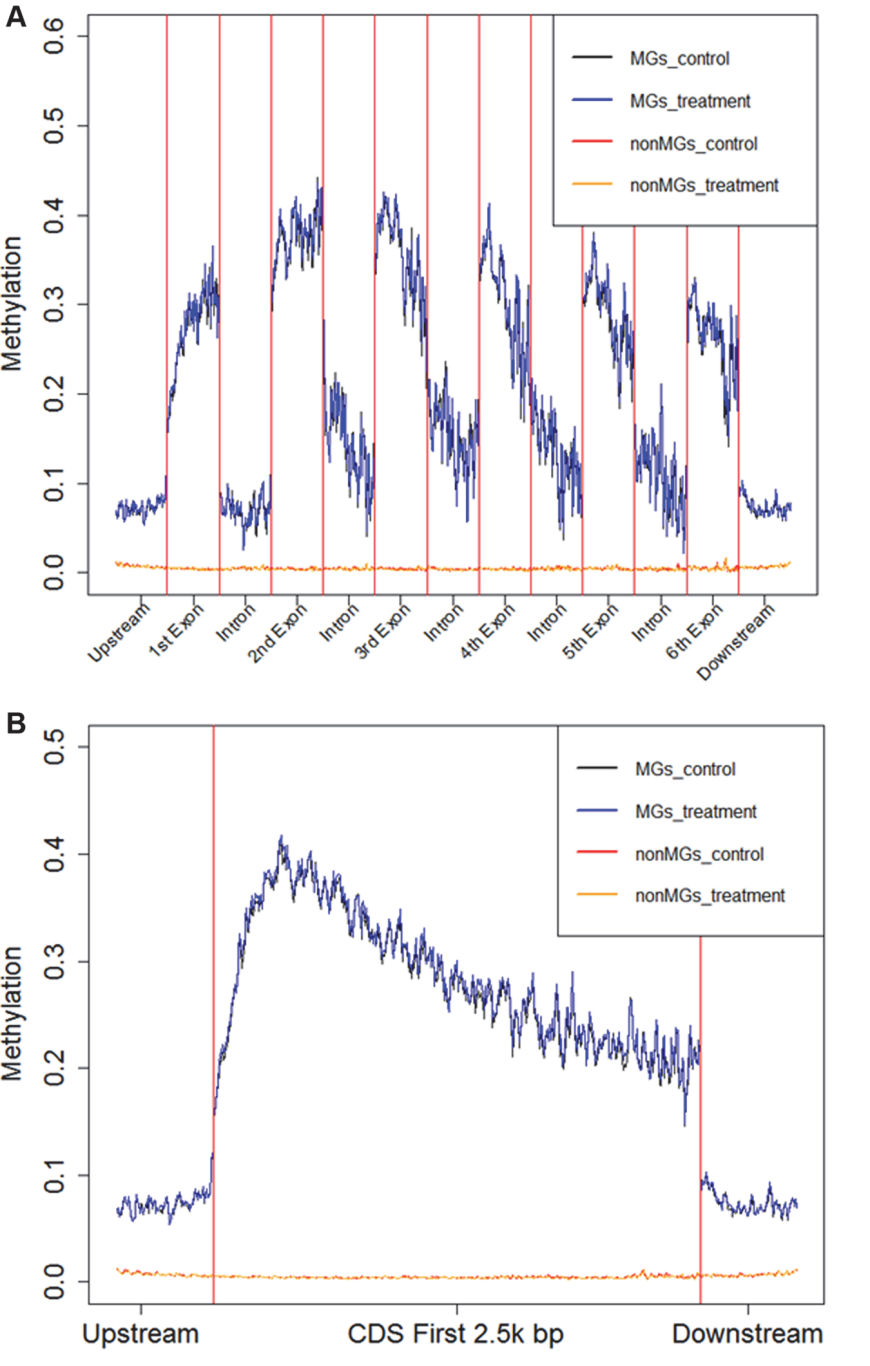
Average fractional methylation levels in different gene regions. **A)** Average Fractional Methylation level for 1kbp upstream of coding region, first 500bp of first 6 exons, first 500bp of first 5 introns and 1kbp downstream of coding region are shown in the figure. **B)** Average Fractional Methylation level for 1kbp upstream of coding region, first 3kbp of protein coding region and 1kbp downstream of coding region are shown in the figure. In this figure, the x-axis is the relative coordinate with a word size of 20bp. The y-axis is the average fractional methylation level of CpGs of all genes in a 20bp word (detailed in Materials and Methods). The black line represents the average fractional methylation level for methylated genes in control and the blue line represents the average fractional methylation level for methylated genes in treatment. The red line represents the average fractional non-methylation level for non-methylated genes in control and orange line represents the average fractional methylation level for non-methylated genes in treatment.

Within a methylated gene, exons are much more heavily methylated than introns, similar to the pattern in *Nasonia vitripennis* [74]. DNA methylation levels peak in the second exon (Fig. 5A) and sharply decrease outside of the coding regions of methylated genes (Fig. 5B). Non-methylated genes are significantly longer than methylated genes (*P* < 10^–16^; Mann-Whitney test), as previously inferred based upon nucleotide composition [75]. Moreover, within methylated genes, gene length is negatively correlated with fractional methylated levels of each exon (Spearman’s correlation coefficient-0.34, *P* < 10^–16^). To exclude the possibility that longer genes appear to be less methylated since DNA methylation is mostly targeted to the 5’ end of genes, we examined the relationship between gene length and DNA methylation using the fractional methylation levels of only the first 500 bps of genes. Gene length is still significantly negatively correlated with methylation level in this analysis (Spearman’s correlation coefficient-0.10, *P* < 10^–15^), indicating that the relationship between DNA methylation and gene (and exon) length is likely to have true biological basis.

### DNA methylation and gene expression

**Fig 6:**
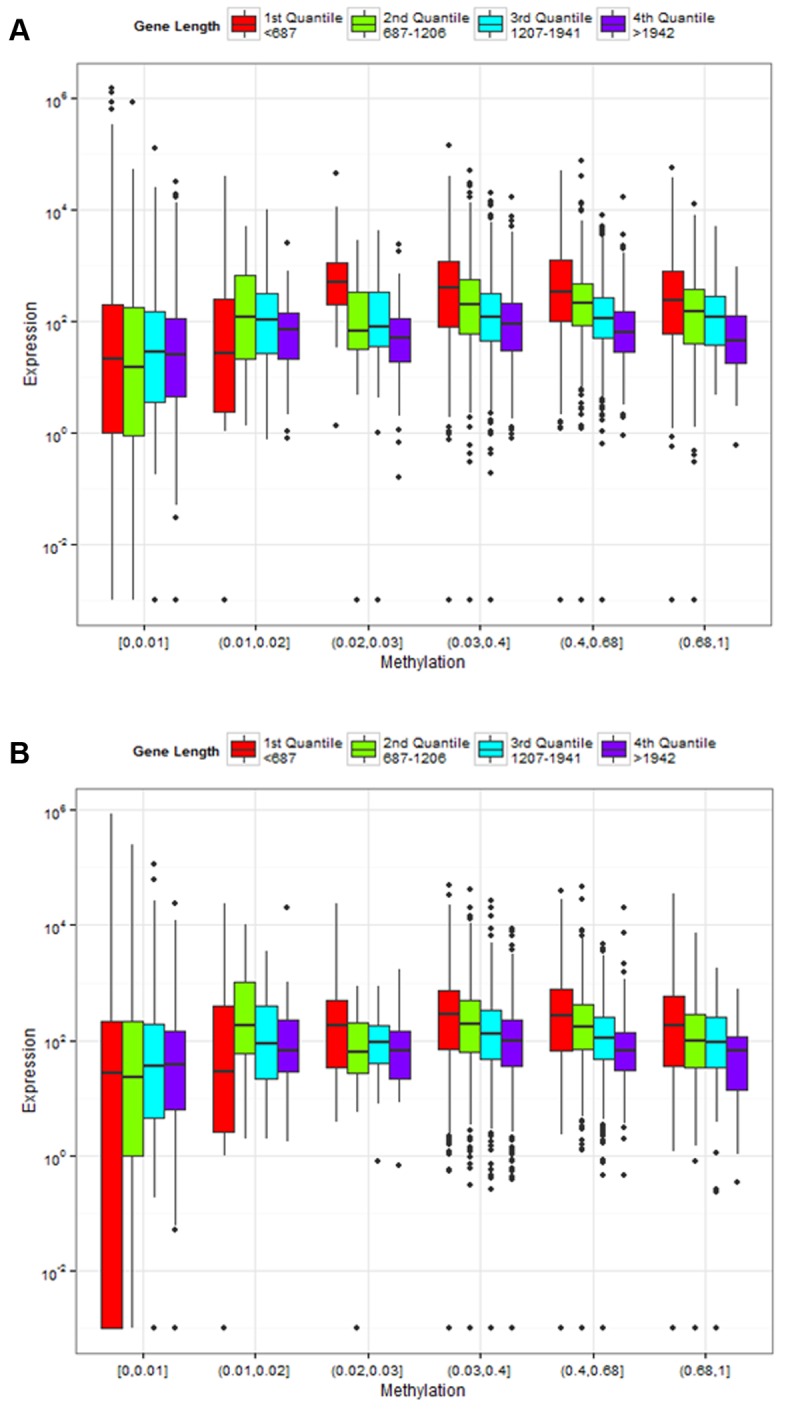
Association of gene length and fractional methylation with gene expression. Relationship between gene expression and DNA methylation is confounded by gene lengths in **(A)** control and **(B)** treatment experiments. The y-axis is the normalized gene expression levels (normalized by TMM methods and then divided by gene length) shown in a log-transformed scale. The x-axis represents fractional methylation levels. The first three groups of genes are sparsely methylated genes divided into three similar sized bins. The last four groups are relatively heavily methylated genes. Methylated genes are first divided into three similar sized bins, and then the most heavily methylated genes (fractional methylation levels > 0.9) are shown separately to represent the relationship between gene lengths and expression more clearly. The last group (fractional methylation level > 0.9) does not include the longest quantile of genes.

We next examined the relationship between DNA methylation and gene expression. Overall, we found that methylated genes are expressed at significantly higher levels than non-methylated genes (*P*<10^–16^, Mann-Whitney test, for both control and treatment, Fig. 6A&B), consistent with the previous studies demonstrating that gene body methylation promotes expression [62,71,76,77]. However, as shown above, DNA methylation is tightly correlated with gene length (longer genes are less methylated). It is also known that gene expression levels co-vary with gene length, in that long genes tend to exhibit reduced expression [78,79]. Fig. 6 demonstrates the interactions between gene length, DNA methylation and gene expression levels, by illustrating the relationship between gene length and gene expression in different methylation bins. First, genes with extremely low levels of DNA methylation are expressed at significantly lower levels than others. For example, genes with methylation < 0.02 have significantly lower expression compared to the rest of the genes for both control and treatment (P < 10^–16^; Mann-Whitney test). Second, gene expression level is strongly associated with gene length. Longer genes generally were expressed at lower levels than shorter genes, except the case of the lowest methylated quantile. Thus, both gene length and DNA methylation affect gene expression, which is also supported by an ANOVA (Table 1). Both control and infected groups exhibited similar trends.

**Table 1:** Gene length and DNA methylation level are associated with gene expression.

**predictor**	**coefficient**	**SE coefficient**	**t**	**P**	
methylation	0.604	0.029	20.64	<10^–16^	
ln(length)	-0.049	0.0058	-8.44	<10^–16^	
treatment	-0.0087	0.014	-0.621	0.535	
Adjusted R^2^ is 0.0.02471					
**ANOVA TABLE**	**Degree of freedom**	**Sum Square**	**Mean Square**	**F value**	**P-value**
Methylation	1	488	488	425.86	<10^–16^
ln(Length)	1	191.2	191.2	166.87	<10^–16^
treatment	1	0.4	0.44	0.385	0.5348
Residuals	23287	26684	1.55		

### Differential DNA methylation as a response to viral infection

We examined the changes of DNA methylation as a response to viral infection by using a generalized linear model where CpG positions are explicitly modeled (Materials and Methods). In a moderately stringent condition of FDR < 0.01, only 40 genes are identified as differentially methylated (S2A Table). Even when we relax the statistical criterion to FDR < 0.1 (a cutoff selected based upon the empirical distributions of P-values to better reflect the data), the number of differentially methylated genes (DMGs) only increases to 156 (S2A Table). Among these 156 putatively differentially methylated genes, 33 of them are more highly methylated in controls while 123 of them are more highly methylated in treatment. Several genes involved in cellular signaling were found to be differentially methylated including five genes that are associated with the T-cell activation pathway in vertebrates, a major immune pathway (S2B&C Table). These genes are inositol 1,4,5,-tris-phosphate receptor (IP3R), phosphotidylinositol 3 kinase (PI3K), protein kinase C-delta (PKC-delta), Vav-like (Vav), and son of sevenless (Sos). All of these genes interact with RNA viruses in human systems. For example, IP3R is required for the release of human immunodeficiency virus (HIV) particles[80]. Inhibition of PI3K negatively affects cell entry of influenza virus and hepatitis C virus[81,82] thereby affecting viral replication. Inhibition of PKC-delta has a negative effect on HIV post cell entry replication[83]. Dominant negative Vav constructs decrease intercellular trafficking of a critical HIV protein[84]. Finally, inactivation of Sos results in an inhibition of protein kinase (PKR) activity, ultimately leading to a resistance to reovirus infection[85]. Unfortunately, information on how these genes respond to viral infection in other species is severely understudied. Though our results and other recent studies have indicated that DNA methylation can be involved in rapid processes—indeed, even learning[86]—it is possible that there are significantly more differentially methylated genes at later time points.

### Differential DNA methylation does not lead to differential gene expression

We then directly examined whether significantly differentially methylated genes (DMGs) are also differentially expressed genes (DEGs), as expected if differential DNA methylation drives the transcriptomic responses to viral infection. In contrast to this prediction, we found that there is little overlap between DMGs and DEGs—only 4 genes were found to be both differentially expressed and differentially methylated, which is not a significant association according to the Fisher’s exact test (*P* = 0.068; 5 genes would be expected to be shared by chance). Among the 4 genes that were both differentially expressed and differentially methylated, three were up-regulated in response to increased DNA methylation, while the other was down-regulated in response to increased DNA methylation. The lack of correspondence between DMGs and DEGs may have been a result of using different sets of bees for each analysis, which was necessary due to the high quantities of RNA and DNA required for these analyses. However, the lack of overlap between DMGs and DEGs was further supported by single CpG level analysis where neither enrichment nor reduction of differentially methylated CpG sites was observed in the 753 differential expressed genes (P = 0.38; Chi-square test).

**Fig 7:**
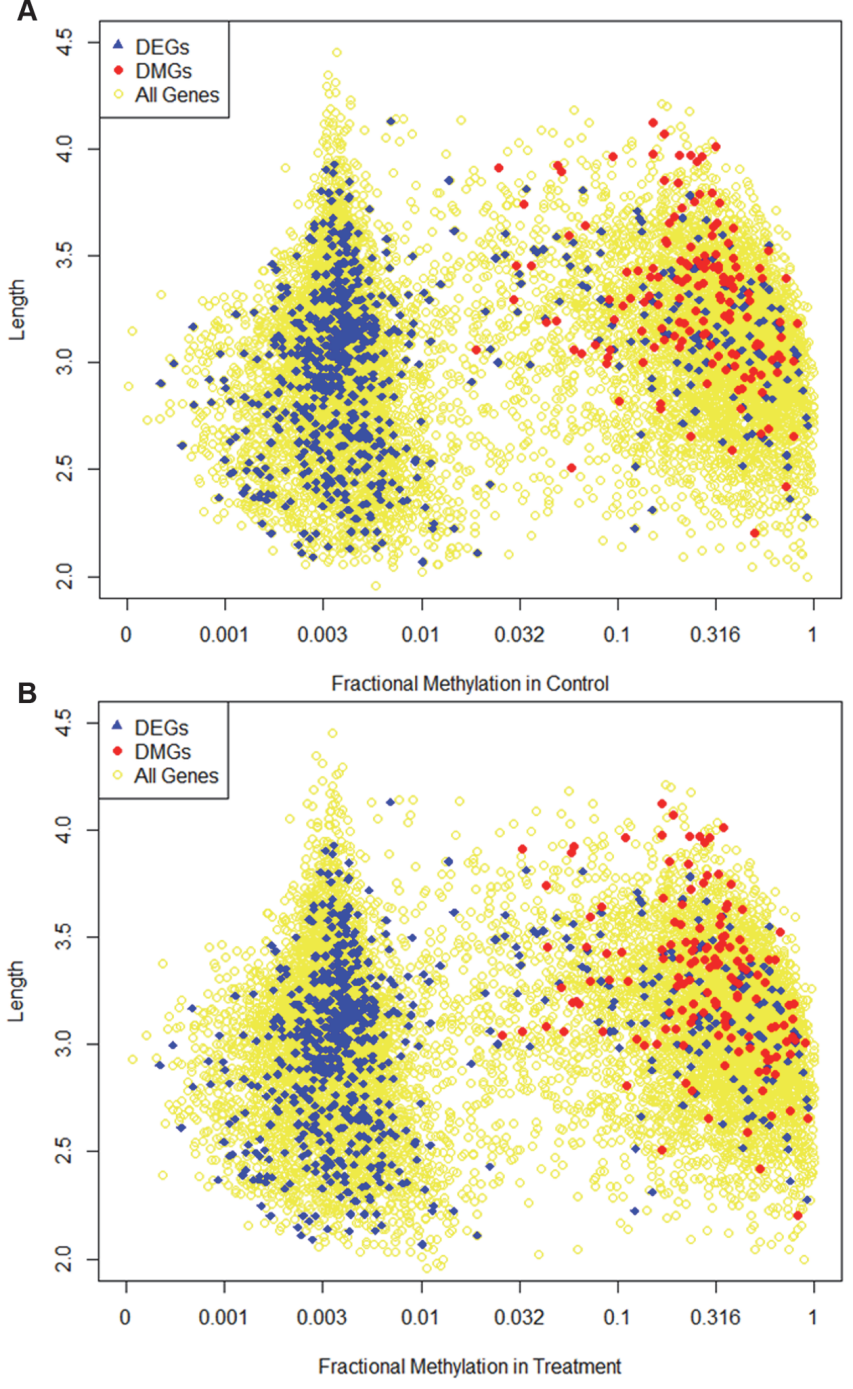
Dispersion of fractional methylation. Different characteristics of differentially methylated genes (DMGs) and differentially expressed genes (DEGs) are shown in the figure. The x-axis is the fractional methylation of genes in control **(A)** or treatment **(B)** shown in log-scale. The y axis is gene length in log-scale. Genes longer than 10000 (1735 genes in total, 53 DEGs, 0 DMGs) are not shown in the figure. The yellow circles represent the background showing the distribution of methylation and length for all genes. Blue triangles represent the DEGs (identical between figures) and red dots represent the DMGs.

In fact, DEGs and DMGs had distinctive characteristics at many levels (Fig. 7). DMGs tend to be highly methylated, and consequently, highly expressed genes (*P* < 10^–10^; Mann-Whitney test). On the other hand, DEGs are significantly less methylated than non-DEGs (mean fractional methylation levels of DEGs is 0.063 compared to 0.197 of non-DEGs, *P* < 10^–16^; Mann-Whitney test). Among the 753 DEGs, 610 of them were classified as non-methylated in both controls and virus-infected bees. DEGs also were highly expressed (*P* < 10^–16^; t test), however, they were not significantly longer or shorter than other genes (median coding region length of DEGs was 1261 as compared to 1203 of non-DEGs, *P* = 0.1, t-test). But coding regions of DEGs are significantly shorter than DMGs (*P* < 10^–16^; Mann-Whitney test). Thus our study suggests that different types of genes respond to biological stimuli in different ways: some genes tend to be differentially expressed, while others tend to be differentially methylated. These two types of genes exhibit different, almost completely opposite, genomic characteristics. However, transcriptional regulation seems to occur at a much greater level in response to viral infection than methylation, with 753 DEGs (FDR<0.05) and only 156 DMGs (FDR <0.1).

### DNA methylation and alternative splicing

Recent studies have demonstrated significant roles of DNA methylation on alternative splicing [43,87–89]. While alternative splicing indicates usage of alternative transcripts, it can also interfere with normal gene function. For example, retaining a non-coding portion of a gene (by intron retention) or missing a crucial exon (exon skipping) in the final transcript may lead to misfolded or non-functional proteins. Thus, DNA methylation changes may reduce gene function via splicing, rather than transcription.

Here we examined the relationship between DNA methylation and alternative splicing (Materials and Methods). We identified 1345 genes with exon skipping (with 90 genes unique to controls, and 43 unique to treatment; S2D&E Table) and 1578 with intron retention (with 81 genes unique to controls, and 156 unique to treatment; S2F&G Table). GO analysis found that genes with exon skipping are enriched in glycerophospholipid metabolic process. Interestingly, glycerophospholipid metabolism plays a key role in flock house virus (FHV, which, like IAPV, is a positive sense RNA virus) replication in *D*. *melanogaster*, where it is involved in FHV RNA replication complex assembly and function[90].

**Fig 8:**
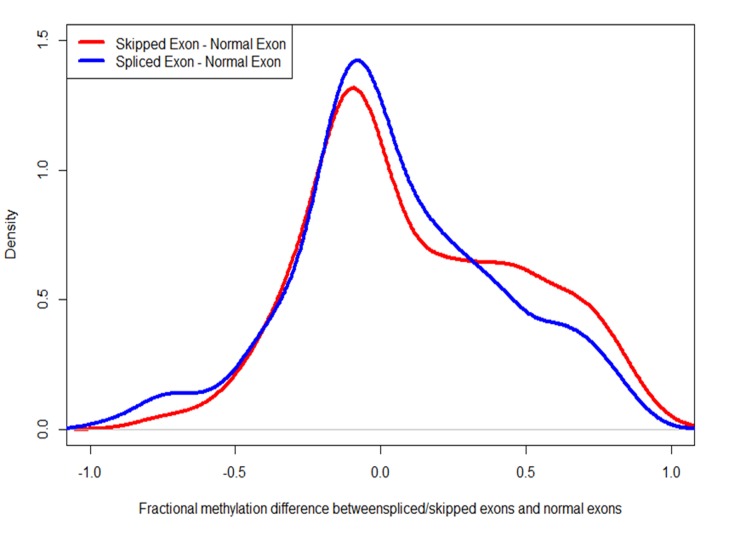
Alternatively spliced exons are more heavily methylated than constitutively used exons. The red curve depicts the difference in the fractional methylation levels of skipped exons (in exon-skipping events) and other exons. The blue curve depicts the difference between spliced exons (in the case of intron-retention events) and constitutively used exons. In both cases, the differences are biased towards positive, indicating that alternatively used exons are more heavily methylated.

We found that exons involved in intron retention are significantly more methylated than other exons in the same genes, in both biological conditions (*P* < 10^–3^, paired Mann-Whitney test). Exons that are skipped in an exon-skipping event show also higher methylation level compared to other exons of that gene (*P* <10^–9^, paired Mann-Whitney test, Fig. 8). However, no significant overlap was observed between those differentially spliced genes and DMGs. Moreover, exons involved in alternative splicing between the control and treatment exhibit little difference in DNA methylation between control and treatment (Table 2).

**Table 2:** Exons involved in alternative splicing exhibit little difference in DNA methylation between control and treatment.

	Unique Intron Retention in control	Unique Intron Retention in treatment	Unique Exon Skipping in control	Unique Exon Skipping in treatment
**Control**	0.186	0.115	0.152	0.081
**Treatment**	0.199	0.117	0.150	0.081

### Conclusion

Here we characterized, for the first time, global gene expression and DNA methylation patterns associated with acute viral infection in an insect species, the honey bee. We demonstrated that several transcriptionally regulated genes are associated with previously identified viral response pathways in insects, including the JAK-STAT pathway, the RNAi pathway, and transcriptional pausing. Note that these last two pathways have not previously been observed to be transcriptionally upregulated in response to viral infection in honey bees, and thus represent intriguing new candidates to consider in the development of viral-resistant honey bee strains or management techniques to mitigate the impact of viruses. We found very little overlap of our gene expression patterns with those identified in previous studies of immunostimulation of adult bees with bacteria[69] and microsprodia[70], and though overlap was greater with studies of viral infection[24,27] (particularly chronic infection of older honey bees with IAPV[21]), these results suggest that immune responses can vary substantially across pathogens, parasites and life stages. We have also demonstrated that epigenetic processes, namely DNA methylation, are altered in response to viral infection in honey bees. Changes in DNA methylation patterns have not previously been described to be an antiviral response in insects, but this is likely due to the fact that most insect molecular immunity studies involve *D*. *melanogaster*, which does not have a functional *de novo* DNA methylation system and has very low global DNA methylation levels. Several of the differentially methylated genes identified in our study are associated with antiviral responses in humans, but have not been associated with immune responses in insects, and thus these represent key genes for future studies of insect immunity. There was no significant overlap in the sets of differentially expressed and differentially methylated genes, and these sets of genes have distinct characteristics (genes that were differentially methylated were longer, more methylated, and less expressed than genes whose methylation patterns did not change). The lack of a significant overlap between differentially expressed and differentially methylated genes suggested honey bees, and perhaps other insects, possess parallel transcriptomic and epigenomic response pathways to viral infection.

## Supporting Information

S1 Fig Confirmation of IAPV replication via presence of IAPV negative strand (Row 1) and confirmation of the IAPV infection via presence of the positive strand (Row 2). RNA was extracted from IAPV-treated individuals. cDNA was created with a Tagged-IAPV-Forward (Row1) or Tagged-IAPV-Reverse primers (Row 2). Both negative and positive strand were than amplified via conventional PCR by the addition of Tag and either IAPV-Reverse (Row 1) or IAPV-Forward primers (Row 2). **A)** IAPV-treated samples used for RNAseq (RNA extracted from thoraces, lanes 2–6), IAPV-infected positive control sample (lane 7), cDNA no enzyme and no primers control (lanes 8 and 9), PCR no primer control (lane 10), PCR IAPV-Forward primer added only (lane 11), PCR IAPV-Reverse primer added only (lane 12), PCR IAPV-Forward and IAPV-Reverse primers added (lane 13), PCR Tag and either IAPV-Forward (Row 1) or IAPV-Reverse (Row 2) primers added (lane 14). Note that lane 14 is to demonstrate that amplification is strand-specificity of the TAG primer, and thus there should be no amplification. 1Kb ladder was loaded into lanes 1 and 15. **B)** IAPV-treated samples used for qRT-PCR (RNA extracted from the fat bodies). IAPV-treated samples used for RNAseq (RNA extracted from thoraces, lanes 2–11), IAPV-infected positive control sample (lane 12), cDNA no enzyme and no primers control (lanes 13 and 14), PCR no primer control (lane 15), PCR IAPV-Forward primer added only (lane 16), PCR IAPV-Reverse primer added only (lane 17), PCR Tag and either IAPV-Forward (Row 1) or IAPV-Reverse (Row 2) primers added (lane 18), PCR IAPV-Forward and IAPV-Reverse primers added (lane 19). 1Kb ladder was loaded into lane 1. Arrows on the right indicate the expected 840 bp product.
(TIF)

S2 Fig Identification of exon skipping and intron retention. Alternative splicing events are identified by compare splice junctions to reference. Splice junctions are summarized by Tophat[53] which use reads whose fractions mapped to different locations to identify splice junction. If a pair of junctions span one or more exons, the exons spanned are considered skipped in exon skipping. If a pair of junctions occur inside an annotated exon, there is intron retention event in reference (introns are spliced in transcripts).
(TIF)

S1 Table Information regarding the transcriptomic analysis, including tables of: A. differentially expressed genes, B. primers and associated references for RT-PCR, C. average Ct values for confirmation of viral loads, D. read counts for viruses found in the RNA-seq data set, E. GO terms of down regulated genes, F. GO terms of up regulated genes, G. overlap with canonical immune genes from Evans et al. 2006[68], H. overlap with D. melanogaster acute antiviral response from Xu et al. 2012[64], I. overlap with previous honey bee immunity studies[21,24,27,68–70], and J. the top 10 most up/down regulated genes. (XLSX)

S2 Table Information regarding the DNA methylation analysis, including tables of: A. differentially methylated genes, B. pathways associated with differentially methylated genes, C. significantly differentially methylated genes involved in the T-cell activation pathway, and D. overlap between differentially expressed and differentially methylated genes. (XLSX)
