# Supplementary figures and images for: Parallel Epigenomic and Transcriptomic Responses to Viral Infection in Honey Bees (Apis mellifera)
Source: PLoS Pathog. 2015 Mar 26;11(3):e1004713. doi: 10.1371/journal.ppat.1004713 (PMC4374888; doi:10.1371/journal.ppat.1004713)

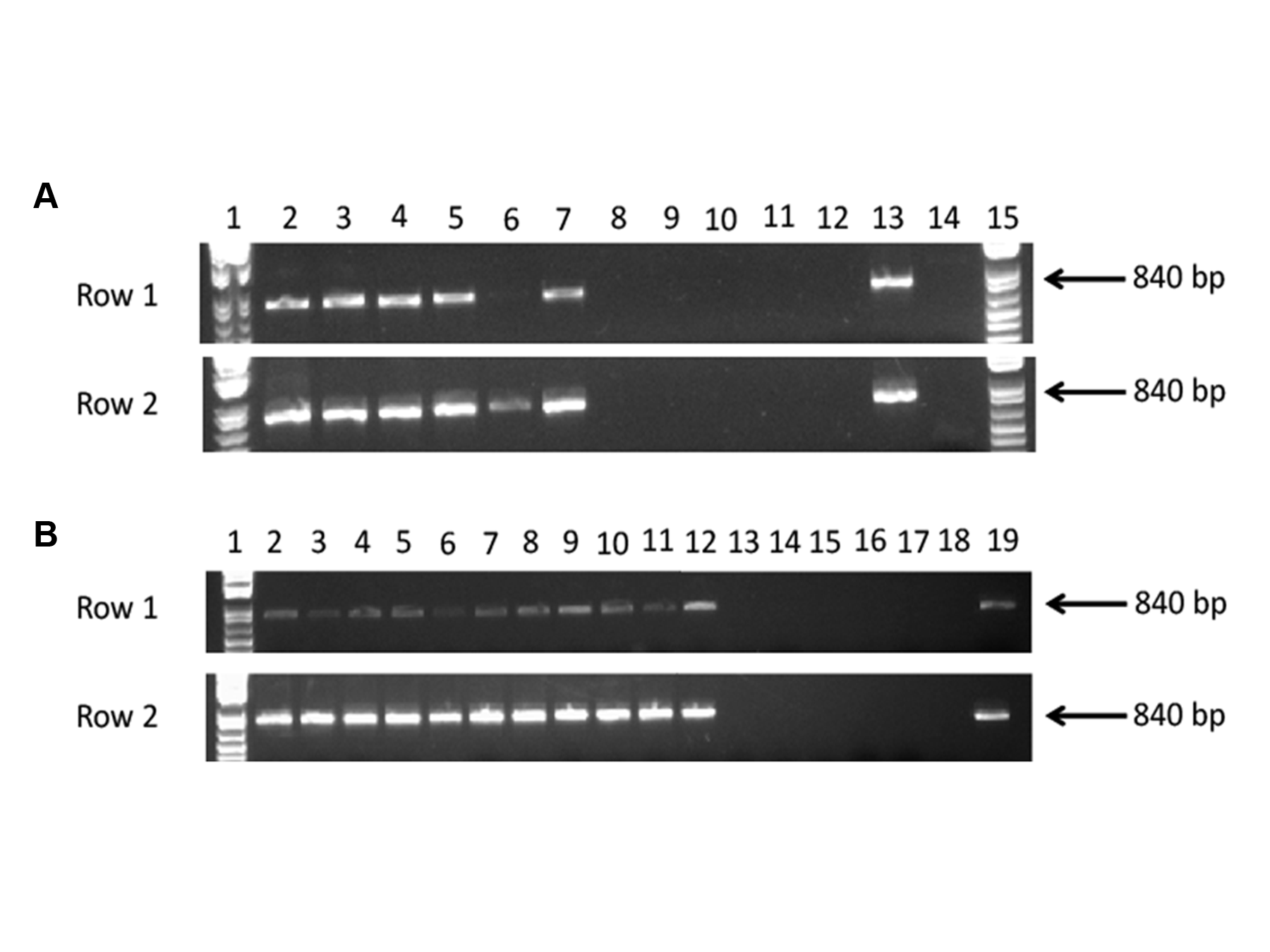

Supplement: S1 Fig — RNA was extracted from IAPV-treated individuals. cDNA was created with a Tagged-IAPV-Forward (Row1) or Tagged-IAPV-Reverse primers (Row 2). Both negative and positive strand were than amplified via conventional PCR by the addition of Tag and either IAPV-Reverse (Row 1) or IAPV-Forward primers (Row 2). A) IAPV-treated samples used for RNAseq (RNA extracted from thoraces, lanes 2–6), IAPV-infected positive control sample (lane 7), cDNA no enzyme and no primers control (lanes 8 and 9), PCR no primer control (lane 10), PCR IAPV-Forward primer added only (lane 11), PCR IAPV-Reverse primer added only (lane 12), PCR IAPV-Forward and IAPV-Reverse primers added (lane 13), PCR Tag and either IAPV-Forward (Row 1) or IAPV-Reverse (Row 2) primers added (lane 14). Note that lane 14 is to demonstrate that amplification is strand-specificity of the TAG primer, and thus there should be no amplification. 1Kb ladder was loaded into lanes 1 and 15. B) IAPV-treated samples used for qRT-PCR (RNA extracted from the fat bodies). IAPV-treated samples used for RNAseq (RNA extracted from thoraces, lanes 2–11), IAPV-infected positive control sample (lane 12), cDNA no enzyme and no primers control (lanes 13 and 14), PCR no primer control (lane 15), PCR IAPV-Forward primer added only (lane 16), PCR IAPV-Reverse primer added only (lane 17), PCR Tag and either IAPV-Forward (Row 1) or IAPV-Reverse (Row 2) primers added (lane 18), PCR IAPV-Forward and IAPV-Reverse primers added (lane 19). 1Kb ladder was loaded into lane 1. Arrows on the right indicate the expected 840 bp product. (TIF) [file ppat.1004713.s001.tif]

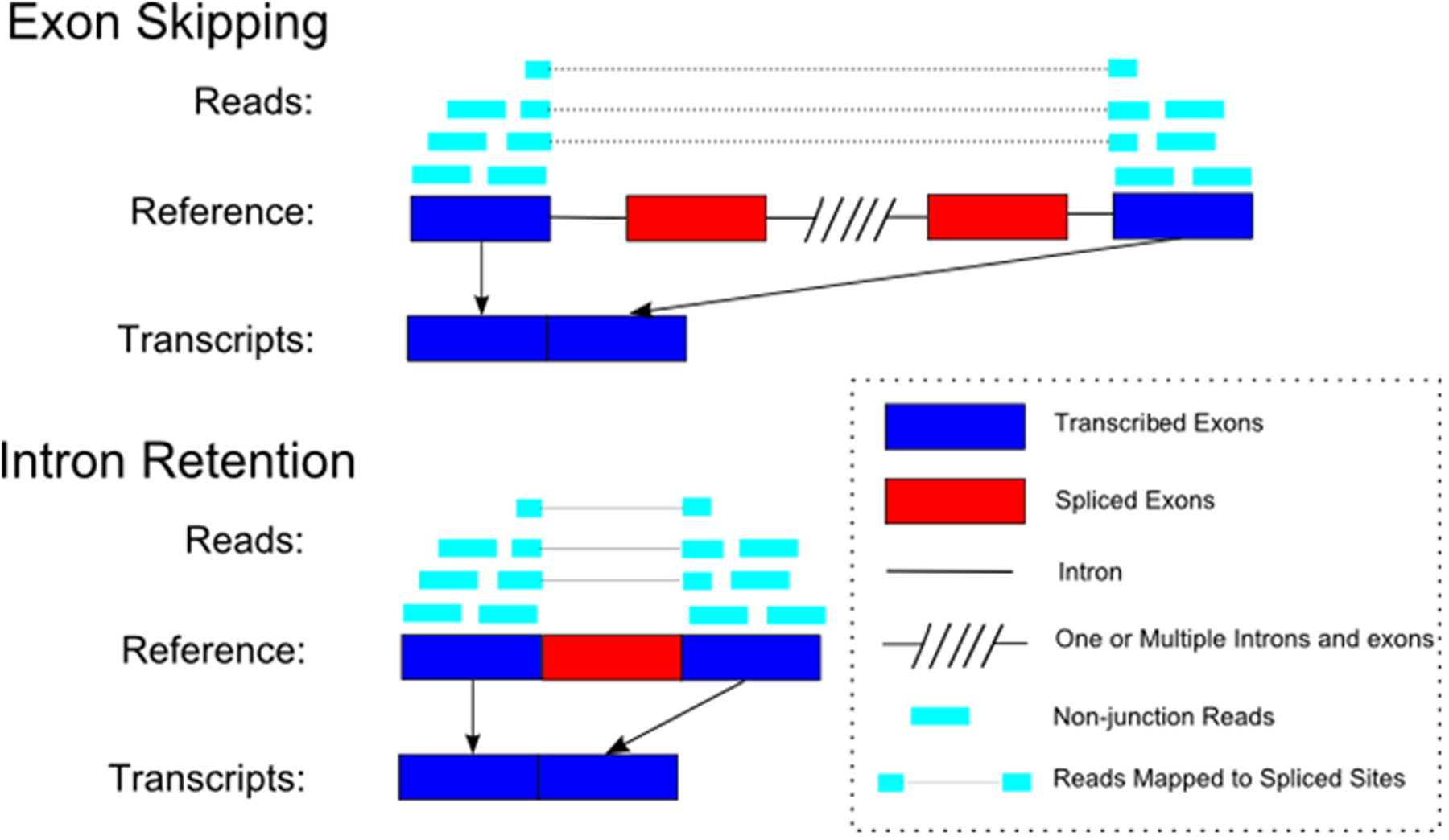

Supplement: S2 Fig — Alternative splicing events are identified by compare splice junctions to reference. Splice junctions are summarized by Tophat[53] which use reads whose fractions mapped to different locations to identify splice junction. If a pair of junctions span one or more exons, the exons spanned are considered skipped in exon skipping. If a pair of junctions occur inside an annotated exon, there is intron retention event in reference (introns are spliced in transcripts). (TIF) [file ppat.1004713.s002.tif]
